# Comparing T- and B-cell responses to COVID-19 vaccines across varied immune backgrounds

**DOI:** 10.1038/s41392-023-01422-7

**Published:** 2023-05-04

**Authors:** Zhen Cui, Wenxin Luo, Ruihong Chen, Yalun Li, Zhoufeng Wang, Yong Liu, Shuo Liu, LeiLei Feng, Zijing Jia, Ruixin Cheng, Jun Tang, Weijin Huang, Yanjun Zhang, Huawen Liu, Xiangxi Wang, Weimin Li

**Affiliations:** 1 CAS Key Laboratory of Infection and Immunity, National Laboratory of Macromolecules, Institute of Biophysics, Chinese Academy of Sciences, Beijing, 100101 China; 2 Department of Respiratory and Critical Care Medicine, Institute of Respiratory Health, Precision Medicine Center, Precision Medicine Key Laboratory of Sichuan Province, Frontiers Science Center for Disease-related Molecular Network, West China Hospital, Sichuan University, Chengdu, 610041 China; 3 Precision Medicine Center, Precision Medicine Key Laboratory of Sichuan Province, Frontiers Science Center for Disease-related Molecular Network, West China Hospital, Sichuan University, Chengdu, 610041 China; 4 Department of Neurology, Chongqing University Three Gorges Hospital, Chongqing University, Chongqing, China; 5 Division of HIV/AIDS and Sex-transmitted Virus Vaccines, Institute for Biological Product Control, National Institutes for Food and Drug Control (NIFDC), No .31 Huatuo Street, Daxing District, Beijing, 102629 China; 6 Department of Microbiology, Zhejiang Provincial Center for Disease Control and Prevention, Hangzhou, China; 7 Department of Oncology, Chongqing University Three Gorges Hospital, Chongqing University, Chongqing, China

**Keywords:** Vaccines, Adaptive immunity

## Abstract

The emergence of adapted variants of the SARS-CoV-2 virus has led to a surge in breakthrough infections worldwide. A recent analysis of immune responses in people who received inactivated vaccines has revealed that individuals with no prior infection have limited resistance to Omicron and its sub-lineages, while those with previous infections exhibit a significant amount of neutralizing antibodies and memory B cells. However, specific T-cell responses remain largely unaffected by the mutations, indicating that T-cell-mediated cellular immunity can still provide protection. Moreover, the administration of a third dose of vaccine has resulted in a marked increase in the spectrum and duration of neutralizing antibodies and memory B cells in vivo, which has enhanced resistance to emerging variants such as BA.2.75 and BA.2.12.1. These results highlight the need to consider booster immunization for previously infected individuals and the development of novel vaccination strategies. The rapid spread of adapted variants of the SARS-CoV-2 virus presents a significant challenge to global health. The findings from this study underscore the importance of tailoring vaccination strategies based on individual immune backgrounds and the potential need for booster shots to combat emerging variants. Continued research and development are crucial to discovering new immunization strategies that will effectively protect public health against the evolving virus.

## Introduction

Since the emergence of SARS-CoV-2 in December 2019, the world has been grappling with a serious health crisis that has affected more than 670 million people and claimed the lives of over 6.8 million people worldwide, according to the World Health Organization. Despite the efforts to control the spread of the virus, new variants of concern (VOCs) have continued to emerge, posing a significant challenge to the global public health response. With the pandemic still spreading, SARS-CoV-2 variants of concern (VOC) have become a major obstacle for us to overcome the outbreak.^1^There are four widely spread VOCs, Alpha, Beta, Gamma and Delta, with numerous reports about them.^2–4^ Lately a newly added VOC, named B.1.529 (Omicron), was found to have a fair number of mutations and produced numerous sub-lineages in the process of spreading.^5–7^ The Omicron sub-variants are possibly the sneakiest, as they usually result in less severe symptoms but have an incredibly high viral load in the upper respiratory tract, making them highly efficient at transmitting the virus.^8^ There were 36 mutation sites in the S protein of Omicron, of which 15 mutation sites located in the receptor binding domain (RBD). In studies of SARS-CoV-2 variants, it was found that RBD mutations lead to increased evasion of vaccine-induced neutralizing antibodies.^9–13^ As Omicron variants continued to spread throughout the world, which gave rise to many sub-lineages (for example BA.2, BA.4, BA.5, BA.2.75). There is mounting evidence indicating that significant alterations in their antigenic characteristics have enabled these Variants of Concern (VOCs) to evade serum neutralization by both vaccinated and convalescent individuals.^14–16^ These variants have different degrees of enhancement in infectivity and immune escape ability, posing a potential threat to the current epidemic prevention and control.^6,17^

In China, various vaccine immunization strategies have been proposed after the outbreak, among which The CoronaVac, a 3-dose β-propiolactone-inactivated vaccine against COVID-19, has been approved for emergency use by the World Health Organization and mass vaccination.^18–21^ Sinovac inactivated vaccine can induce a good humoral immune response and effectively reduce the infection rate, severe rate and mortality.^18^ With emerging VOCs, the broad spectrum of vaccines is increasingly important. Omicron variants have been reported to have potent immune evasion against vaccine-induced neutralizing antibodies,^7,20,22,23^ which may increase the risk of breakthrough infection. Increasing evidence also supports the crucial role of the T-cell response to SARS-CoV-2 in controlling the disease. Studies have shown that higher counts of CD8+ T cells in the lungs are associated with better control of SARS-CoV-2 progression.^24^ Moreover, the presence of T follicular helper cells and CD8+ T cells with activated phenotypes in the blood at the time of virus clearance suggests an active role in the immune response of recovered patients.^25^ According to previous research reports, the Omicron variant has a strong ability to escape humoral immunity mediated by B cells by changing key amino acid sites on the RBD and NTD, but T-cell epitopes are relatively conserved.^26^ Therefore, we are very concerned about the resistance of vaccinated individuals and previously infected individuals to variants, as well as investigating whether strengthening immunity can increase neutralizing antibody titers and specific cellular responses in the body.

To address this issue, our team conducted a study to explore vaccine-induced immune responses to different variants at the antibody and cellular levels.^21,27^ Here we recruited 59 volunteers, and divided them into a natural infection group (prior infection) and a healthy group (naive). Both groups were vaccinated with two or three doses of CoronaVac inactivated vaccine. The levels of neutralizing antibodies in serum and T/B-cell responses were measured at specific times.^28,29^ Our results showed that Omicron and its sub-lineages were capable of immune evasion under the two-dose vaccine immunization strategy compared with other variants. Fortunately, the level of neutralizing antibodies against Omicron and its sub-lineages increased significantly in the prior infection group after booster immunization, suggesting that boosting immunity in the presence of a strong immune memory can effectively improve the body’s resistance to VOCs. The T-cell responses that are induced by various variant S proteins share similarities. A third vaccine dose as a booster will enhance the duration and breadth of antibody response. These findings have important implications for the development of effective vaccination strategies against emerging VOCs, especially in individuals who have previously been infected with SARS-CoV-2.

In conclusion, the emergence of new variants of SARS-CoV-2 presents a major challenge to global public health. The continued spread of the epidemic, the emergence of VOCs, and the potential for breakthrough infections in previously infected individuals highlight the urgent need for effective vaccination strategies that can provide broad protection against emerging variants. Our study demonstrates the importance of booster immunization in individuals with prior infection and suggests that the development of new vaccines or modifications to existing vaccines may be necessary to combat emerging variants.

## Results

### VOCs mutation sites and immune strategies

**Fig. 1:**
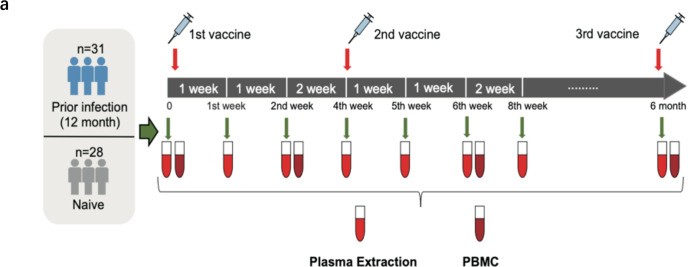
Immune strategies. **a** Vaccination schedule. Vaccinees were stratified into two subgroups: recovered patients (*n* = 31) with prior infections 12 month ago and healthy individuals (*n* = 28), both inoculated two or three doses of CoronaVac inactivated vaccines. The red arrows indicate the time of vaccination; the green arrows indicate the time points at which blood was collected. PBMCs and plasma were isolated for subsequent experiments

It is well known that 2019-nCoV is a single-stranded RNA virus. Due to this characteristic, the virus is very easy to produce adaptive variants under screening pressure. At present, there are more than 1800 kinds of Pango lineages,^30^ and the variants with significantly improved infectivity, immune escape ability and pathogenicity have been listed in VOC by World Health Organization (WHO). For Gamma, Beta, Delta and the newly listed Omicron, a large number of mutations appeared in the S protein, especially Omicron and its sub-lineages such as BA.5 and BA.2.75 (Table 1). Surprisingly, there are more than 30 mutation sites in the S protein of the Omicron. These mutations may be related to virus stability, immune escape and infectivity. Omicron has become a new epidemic strain, and many lineages have been derived in the process of transmission. To investigate B and T-cell immune responses against circulating VOCs, we recruited and grouped 59 volunteers from West China Hospital Sichuan University, Chengdu or Three Gorges Hospital Chongqing University, Chongqing. Thirty-one volunteers who were previously infected with WT SARS-CoV-2 were in the prior infection group, and the other 28 volunteers were in the naive group who were not infected with the virus. In the 0th week and the 4th week, the two groups of volunteers were vaccinated with CoronaVac inactivated vaccine. Additionally, ten volunteers received a third dose of vaccine around the 6th month after the second administration. Baseline characteristics of the participants were similar across the treatment groups (Table 1). Their bloods were then collected at 0, 1, 2, 4, 5, 6 and 8 weeks as well as 6 months to isolate serum and PBMCs (Fig. 1a). There were no significant differences in the prevalence of any solicited or unsolicited reactions between the two groups.

**Table 1:** The basic information of the participants and the mutation sites of VOCs

	Previous SARS-CoV-2 infected group (*n* = 31)	Normal healthy control group (*n* = 28)
Age, years		
18–29	7 (22.6%)	7 (25.0%)
30–39	7 (22.6%)	8 (28.6%)
40–49	10 (32.3%)	8 (28.6%)
50–60	7 (22.6%)	5 (17.9%)
Mean age, years	39.1 (10.3)	38.9 (10.7)
Sex		
Male	20 (64.5%)	16 (57.1%)
Female	11 (35.5%)	12 (42.9%)
Han ethnicity	31 (100.0%)	28 (100.0%)
Mean body mass index, kg/m^2^	23.7 (3.2)	23.2 (3.0)
Underlying diseases^a^		
Yes	6 (19.4%)	3 (10.7%)
No	25 (80.6%)	25 (89.3%)

Data are *n* (%) or mean (SD)

^a^Nine participants had hypertension, type 2 diabetes, asthma, IgA nephropathy, or were a carrier of hepatitis B virus

Alpha: Δ69–70, Y144del, N501Y, A570D, D614G, P681H, T716I, S982A, D1118H

Delta: T19R, G142D, EF156–157del, R158G, L452R, T478K, D614G, P681R, D950N

Omicron: A67V, Δ69–70, T95I, G142D, Δ143–145, Δ211, L212I, ins214EPE, G339D, S371L, S373P, S375F, K417N, N440K, G446S, S477N, T478K, E484A, Q493K, G496S, Q498R, N501Y, Y505H, T547K, D614G, H655Y, N679K, P681H, N764K, D796Y, N856K, Q954H, N969K, L981F

### S protein-specific antibody responses of the volunteers after vaccination

**Fig. 2:**
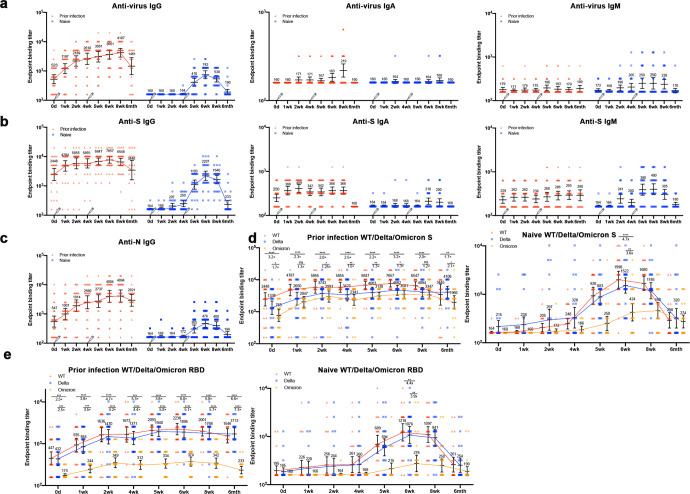
S protein-specific antibody responses of the volunteers after vaccination**. a** IgG (left), IgA (mid), and IgM (right) endpoint antibody responses specific to total virus of wide type measured with ELISA at 0 wk, 1st wk, 2nd wk, 4th wk, 5th wk, 6th wk, 8th wk and 6th mth after first dose. Plasma samples were collected from participants (prior infection of WT strain: *n* = 31; naive: *n* = 28) inoculated two doses of CoronaVac inactivated vaccines at baseline. Black bars and indicated values represent geometric mean values in 95% confidence intervals (CIs); Each point represents a single individual; the red points represent prior infection individual and the blue represent naive. **b** IgG (left), IgA (mid), and IgM (right) endpoint antibody responses specific to spike (S) protein of wide type at 8 time points as described before. **c** IgG endpoint antibody responses specific to nucleocapsid (N) protein of wide type at 8 time points. **d**, **e** Endpoint antibody responses specific to the spike and RBD of WT, Delta, and Omicron VOCs from recovered (left) and naive vaccinees (right). Antibody endpoint binding titers fold change for Delta or Omicron to wild-type for each group of sera is shown in each of the plots

We collected blood samples from the prior infection and naive groups at the indicated time points, and isolated serum and PBMC. To monitor the dynamics of three types of antibodies in sera, we performed enzyme-linked immunosorbent assay (ELISA) to measure the binding titers of each type antibody (IgG, IgA, IgM) in these sera by using both intact virions and S proteins (Fig. 2a, b, supplementary Fig. 1). As expected, IgG against SARS-CoV-2 was present in all samples in the Prior infection group before vaccination, but IgA and IgM were not detected. SARS-CoV-2-specific IgG levels surged in vaccinees previously infected with SARS-CoV-2 one week after the first dose (week 1) and continued to rise within four weeks after the first dose, with one exception followed by almost unaltered antibody titers. On the contrary, the levels of IgA and IgM did not change significantly after the first administration. In addition, no detectable SARS-CoV-2-specific IgG emerged within four weeks after the first injection in naive group, highlighting distinct immune magnitude of individuals from two groups towards the first vaccination (Fig. 2a). After the second administration of the two groups, the virus-specific IgG of the prior infection group continued to rise, and the naive group increased significantly and reached a peak at the 6th week (14 days after the second dose), which was similar to the level of 12 months after natural infected. For virus-specific IgM and IgA, there was no overall trend as IgG. To assess whether vaccine responses were limited to virus or could be extended to antibody responses against the S protein and N protein, we also used ELISA to measure anti-S protein and N protein IgG, IgA, and IgM in serum (Fig. 2b, c) . S protein-specific IgG changes similar to virus-specific IgG. To compare the antibody titers of the vaccinated sera to different variants, we analyzed the affinity of the volunteers’ sera at 8 time points to the S protein of each variant by ELISA. The antibodies against Beta, Gamma, Delta and especially Omicron variants decreased at different degrees, compared with wild-type S protein. The significant decrease in the antibody titer of Omicron S protein may be due to the existence of multiple mutation sites in the important domain of Omicron S protein (Fig. 2d, supplementary Fig. 2). Considering the important role of RBD in virus invasion and also is an important domain of virus immune escape, we detected the specific antibodies against each variant RBD. Surprisingly, even for the prior infection group (6 weeks) who had higher affinity, the reduction of Omicron RBD-specific antibodies was incredibly significant. Omicron RBD geometric mean titers decreased from 2238 and 1895 to 374 compared to WT and Delta, respectively (*p* < 0.0001). An additional 1/3 of the volunteers had Omicron RBD antibody titer below the detection limit (Fig. 2e). The above results showed that CoronaVac inactivated vaccine can induce good humoral immune responses in both prior infection and naive groups, but the two-dose vaccine immunization strategy may not be sufficient to against Omicron and its sub-lineages infection.

### Neutralizing antibody titers against SARS-CoV-2 variants

**Fig. 3:**
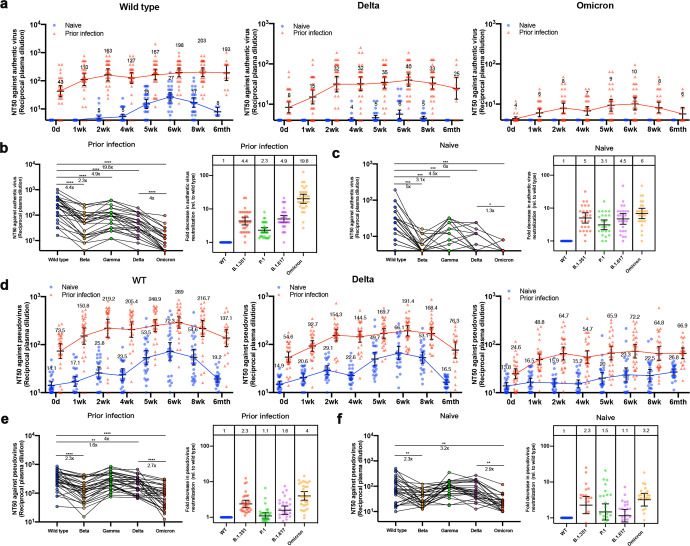
Neutralizing antibody titers against live SARS-CoV-2 variants. Plasma neutralizing activity evaluated by authentic SARS-CoV-2 (**a**) and pseudotyped SARS-CoV-2 neutralization assays (**d**). Half-maximal neutralizing titer (NT50) values for sera from cohorts collected at 8 time points described above against SARS-CoV-2 WT (left), Delta (mid), and Omicron (right). Left: Authentic virus (**b**) and pseudovirus neutralization assays (**e**) are plotted for cohorts (prior infection of WT strain: *n* = 31) 2 weeks out from second dose of CoronaVac for wild-type, Beta, Gamma, Delta and Omicron. the indicated values represent NT50 values fold change for each variant relative to wild-type for each cohort of plasma samples. ****p < 0.0001. Left: Authentic virus (**c**) and pseudovirus neutralization assays (**f**) are plotted for cohorts (naive: *n* = 28) 2 weeks out from second dose of CoronaVac for wild-type, Beta, Gamma, Delta and Omicron

Neutralizing antibody (NAb) is the main reason for the immune protection of the body against many viruses, and has a good correlation with the protective effect of vaccines. Thus we measured neutralizing antibodies in sera from 59 volunteers against WT, P.1, B.1.351, B.1.617 and B.1.529 authentic SARS-CoV-2 and the corresponding pseudovirus. The geometric mean half-maximal neutralizing titers (GMT NT50) against authentic SARS-CoV-2 in plasma obtained from the prior infection and naive groups suggested that a certain amount of NAb existed in the serum before vaccination in prior infection group. The NAbs of prior infection group rapidly increased after one dose while they didn’t increase in the naive group until two injections were finished. And the NAb levels of both groups reached the maximum at 6 weeks, and the trends were similar among different variants (Fig. 3a). When analyzing neutralizing antibodies of prior infection group (*n* = 31, 6th weeks) (Fig. 3b), taking WT as a reference, the levels of NAbs against Beta, Gamma, Delta and Omicron decreased by 4.4-fold, 2.3-fold, 4.9-fold and 19.8-fold, respectively. Compared to Delta, NAbs against Omicron is down 4 times. There were 12 volunteers whose Omicron-specific NAbs were undetectable. Neutralizing antibodies in the naive group (*n* = 28 6th weeks) also were analyzed (Fig. 3c). Notably, 26 volunteers had their Omicron-specific NAbs below the limit of detection. Further using the VSV pseudovirus expressing WT/Delta/Omicron S protein, the GMT NT50 of the prior infection group (6 weeks) were 289, 191 and 72, respectively, and the GMT NT50 of the Naive group were 72, 66 and 23, respectively (Fig. 3d), which were 2–7 times higher than the results measured by authentic SARS-CoV-2 suggesting higher sensitivity of neutralizing antibody titers based on pseudovirus detection. Consistent with the results of authentic SARS-CoV-2 neutralization assays, NAb levels against Beta, Delta, and Omicron were reduced by 2.3-fold, 1.6-fold, and 4-fold in the previously infected group, respectively (Fig. 3e, f). Similarly, the levels of NAbs against Beta and Omicron of the Naive group decreased by 2.3 times and 3.2 times. Gender and age did not affect significantly neutralizing titers in our cohort (supplementary Fig. 3). For Omicron, the breadth of the existing vaccines is obviously far from enough. With the prevalence of Omicron, breakthrough infections are highly likely, even for the recovered patients. To sum up, the humoral immunity of healthy people after receiving two doses of inactivated vaccine (6 weeks) and before vaccination (0 week) of recovered patients severely decreased when confronted with the mutant strains, especially Omicron. To prevent breakthrough infections caused by the mutant strains such as Omicron and its sub-lineages, under the existing immunization strategy, it may be necessary to boost the immunization. For example, after boosting the wild-type inactivated vaccine in the prior infected group, the neutralizing antibodies of each variant were significantly increased, this indicates that boosting immunization can effectively improve the broad spectrum of antibodies.

### Omicron escapes SARS-CoV-2-specific B cells mediated long-lasting immunity

**Fig. 4:**
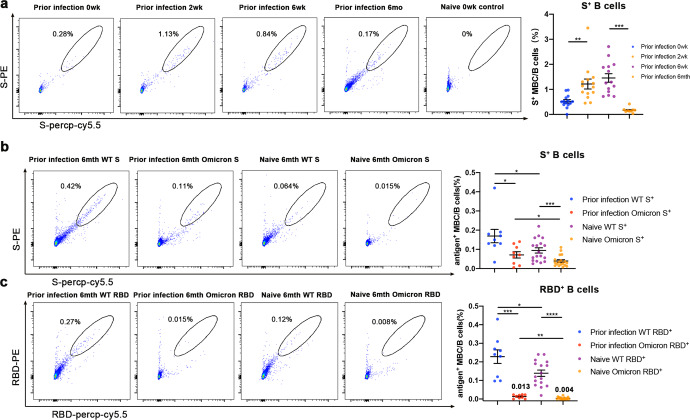
Omicron escapes SARS-CoV-2-specific B cells mediated long-lasting immunity. **a** Peripheral Blood Mononuclear Cells (PBMC) were isolated from recovered individuals at four time points: 0, 2, 6 weeks and 6 months after first dose, and naive individuals (*n* = 28) 0 d before first dose as negative control. Representative flow cytometry plots showing dual S-percp-cy5.5 and phycoerythrin (PE)-S-binding memory B cells and graphs indicate the percent of S-binding memory B cells in the B cells. **b**, **c** Comparative memory B cells in PBMCs isolated from study individuals 6 months out from first dose in response to wild-type and Omicron S protein and RBD

As shown by our previous data, total antibodies and neutralizing antibodies peaked 14 days after the volunteers received the second dose. Subsequently the levels of antibodies continued to decline over time, which did not mean the loss of immune protection however. On the contrary, if the virus invades the body within a certain period of time, the immune system will quickly produce specific antibodies against it. As we can see, immune responses of the prior infection group were rapidly launched after one dose (Figs. 2a, b and 3a). This kind of quick immune response is mediated by specific memory B cells (MBCs). To verify the long-acting and broad-spectrum specificity of MBCs, we collected blood from the prior infection group (*n* = 14) at 0, 2, 6 weeks and 6 months of immunization to isolate PBMCs, and then measured their MBCs through flow cytometry (FACS). Changes in S protein-specific B cells^27,31,32^(CD3^−^CD19^+^CD20^+^S^+^) were obvious (Fig. 4a, supplementary Fig. 4). Consistent with previous data, S protein-specific B cells (0.52% of total B cells) were present in the prior infection group at week 0, which explained the more rapid immune response in the prior infection group at the antibody level. S protein-specific B cells increased significantly at second week (1.21% of total B cells), sixth week (1.46% of total B cells) and the proportion of specific B cells decreased after 6 months (0.17% of total B cells). The specific B cells at 6 months after immunization in the naive group (*n* = 18) was 0.094% (Fig. 4b). The proportion of specific MBC could show the potential of immune protection to a certain extent. When MBC continues to decline, it may be necessary to boost immunization to maintain this memory. In order to more comprehensively evaluate the immune responses after vaccination, we analyzed the S protein-specific T-cell response (IFNγ-secreting cells) with the enzyme-linked immunospot assay (ELISPOT) at 0, 2, and 6 weeks after the volunteers were vaccinated.^26,33–36^ It was shown that the proportion of S protein-specific T cells in the prior infection group increased rapidly after the first injection, and both the naive and prior infection group could induce good T-cell responses after two doses (supplementary Fig. 5). Further we compared the previously infected group to WT, Delta, and Omicron variant specific T-cell responses at two weeks after the second dose of the vaccine, and our analysis found that different variants induced similar T-cell responses,^37^ the lack of significant difference may also be related to the relatively small sample size. Given that total antibodies and neutralizing antibody titers were significantly decreased for Omicron, we speculated that the proportion of specific B cells might also be decreased. Through flow cytometry we compared the specific B cells recognizing WT and Omicron S/RBD in prior infection (*n*=9, 6mth) and naive (*n*=18, 6mth) groups (Fig. 4b, c). Omicron S protein-specific B cells decreased significantly in both groups. Statistically, they decreased from 0.17%, 0.094% to 0.071%, 0.039%. As for Omicron RBD-specific B cells, they decreased from 0.23%, 0.14% to 0.013%, 0.004%, respectively. The above results show that mutations in the S protein, especially the RBD, are an important reason for the immune escape of the virus. To sum up, boosting immunization in the presence of existing immune memory (such as the previously infected group) can effectively increase the level of specific T/B cells against different variants, and the variants have a weaker ability to escape T cells. Therefore, in order to improve the long-acting and broad-spectrum immune protection, a third dose or sequential booster immunization may be necessary 6 months after the first dose.

### The third dose of vaccination increases the persistence and broad-spectrum of humoral immunity

**Fig. 5:**
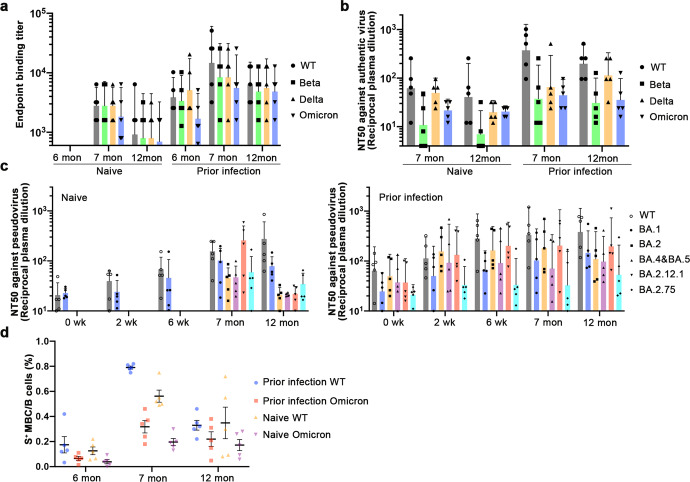
The third dose of vaccination increases the persistence and broad-spectrum of humoral immunity. **a** Plasma samples were collected from participants (prior infection *n* = 5; naive *n* = 5) inoculated two doses of CoronaVac inactivated vaccines at baseline. Each point represents a single individual. **b** Half-maximal neutralizing titer (NT50) values for sera from cohorts collected against authentic virus SARS-CoV-2 of WT, Beta, Delta, and Omicron. **c** Pseudovirus neutralization assays are plotted for naive volunteers (left) and prior infection (right) 0 wk before, 2nd wk, 6th wk, 7th and 12th mth after first dose CoronaVac for each of the following SARS-CoV-2 live viruses: wild-type, BA.1, BA.2, BA.3, BA.4&5, BA.2.12.1, and BA.2.75. **d** Peripheral Blood Mononuclear Cells (PBMC) were isolated from individuals (*n* = 5) at 6th months, 7th months, and 6 months after first dose. Representative flow cytometry plots showing dual S-percp-cy5.5 and phycoerythrin (PE)-S-binding memory B cells of wild type and Omicron respectively

With the emergence of more and more SARS-CoV-2 variants, especially the prevalence of some variants with significantly enhanced immune evasion (BA.4, BA.5B, A.2.75, etc.). The strategy of two doses of inactivated vaccine immunization is not sufficient to provide adequate immune protection for the general population, so a third booster dose is currently being promoted. Here we explored the persistence and broad spectrum of immune responses following a third booster dose in people with different immune backgrounds. One month after inoculation of the third dose, the titers of specific antibodies against each variant S protein in the serum of the two groups were significantly increased, and broad spectrum increased of antibodies. For the prior infection group, WT, Delta and Omicron S protein-specific antibodies increased from 7657, 4682 and 3501 to 14703, 8445 and 5572, respectively, 14 days after the second dose of vaccine and one month after three doses. For the Naive group, WT, Delta and Omicron S protein-specific antibodies increased from 1998, 1552 and 424 to 2786, 2785 and 1838, respectively, 14 days after the second dose of vaccine and one month after third doses (Figs. 2d and 5a). On the other hand, the maintenance time of specific antibodies in the body increased after the third injection of the two groups. For the prior infection group, WT and Omicron S protein-specific antibodies increased from 3880 and 1688 to 6400 and 4850 at 6 versus 12 months after the first dose of the vaccine. For the naive group at sixth month after the first dose of the vaccine specific antibodies were all below the lower limit of detection, with significant increased at 12 month (Figs. 2d and 5a). We further detected neutralizing antibody levels at different time points after third injections (Fig. 5b, c), which were similar to the results of S protein-specific antibodies. In order to evaluate the resistance against the Omicron variant lineage after the third dose of vaccine in the two groups, we used pseudovirus to detect serum against BA.1, BA.2, BA.4&BA.5, BA.2.12.1 and BA.2.75 of neutralizing antibodies. These results showed that the level of neutralizing antibodies against the Omicron sub-lineages could be effectively increased after the third dose of the prior infection group, and the level of neutralizing antibodies could still be maintained at a high level 6 months after the third dose (Fig. 5c). The third booster immunization of the prior infection group with stronger immune memory could more effectively enhance the resistance to the Omicron variant lineage. On the other hand, we compared the proportion of MBC specific for WT and Omicron S protein before and after the third injection in the two groups. It was found that the proportion and duration of MBC could be effectively increased after the third dose of vaccination for people with different immune backgrounds. The MBCs against WT and Omicron in the previously infected group increased from 0.175% and 0.065% (6 mon) to 0.79% and 0.318% (7 mon) after the third dose of inoculation, and the MBCs remained at a high level (0.33% and 0.22% (12 mon)) 6 months after inoculation. The changes of specific MBC in the naive group were similar to those in the prior infection group. The specific MBC for WT and Omicron S protein increased from 0.126% and 0.039% (6 mon) to 0.562% and 0.196% (7 mon). It remained at 0.349% and 0.172% (12 mon) after 6 months of the third dose (Fig. 5d). In conclusion, boosting immunization in the presence of existing immune memory can effectively improve the persistence and broad-spectrum of neutralizing antibodies in serum and specific MBCs in PBMCs of the two groups.

## Discussion

After the outbreak of SARS-CoV-2, the world has been grappling with the COVID-19 pandemic, which has claimed millions of lives and disrupted economies and societies across the globe. Fortunately, with the increase in the global vaccine penetration rate, the epidemic has been effectively controlled to some extent. However, the emergence of new variants of concern (VOCs) has become a major challenge for vaccine design and immunization strategies. Among them, Omicron has been listed as a VOC after its first appearance in South Africa and has rapidly spread around the world due to its strong immune evasion ability.

As a result of Omicron’s strong immune evasion ability, it is highly likely to cause breakthrough infections in recovered and vaccinated populations.^20,21^ Therefore, it is imperative to develop new immune strategies and therapeutic targets to combat this emerging threat. One possible solution is to boost the immune response in recovered and vaccinated individuals.

For convalescent individuals who have developed a strong immune memory, boosting their immunity through immune-inactivated vaccines can effectively enhance the levels of neutralizing antibodies and broad-spectrum immunity. Furthermore, boosting the immune response can elevate the secretion of IFN-γ T cells, reducing the risk of breakthrough infections by emerging variants. In this study, we aimed to investigate the immune response of individuals with different immunological backgrounds after receiving inactivated vaccines, and to identify new immune strategies and therapeutic targets to combat the emerging threat of COVID-19.

In this study, volunteers who had recovered from COVID-19 one year prior and unvaccinated volunteers were recruited and vaccinated according to the immunization strategy mentioned earlier. The analysis found that all participants reached maximum levels of total antibodies and neutralizing antibodies at 6 weeks. Furthermore, the levels of the naive group (6 weeks) and the prior infection (0 weeks) were comparable, indicating that the antibody levels in the naive group after two doses of immunization were similar with those after one year of recovery.^38^ The total antibodies and neutralizing antibodies against different variants Beta, Gamma, Delta and Omicron were measured.^39^ The analysis found that the immune evasion ability of Omicron was prominant compared to other variants.^40,41^ Although the total antibodies and neutralizing antibodies against Omicron in the Prior infection group decreased significantly compared with WT, the GMT NT50 (6week) decreased from 289 to 72. However this decrease was acceptable because there were still a large amount of Omicron-specific antibodies in the serum. And the neutralizing antibodies against Omicron increased significantly at 0 and 6 weeks before (24.6) and after (72.2) vaccination. In addition, enhancing immunity can effectively boost the T-cell response of S protein-specific IFN-γ secretion in terms of specific T-cell responses.^42,43^ For convalescent patients, a single dose of inactivated vaccine can significantly elicit S protein-specific T-cell responses (5.5/10^6^ cells to 46.1/10^6^ cells), and there is no significant difference in the response to the S protein of the wild-type, Delta, and Omicron strains, with similar levels of IFN-γ-secreting T cells. After receiving the third dose of vaccine to boost their immunity, convalescents showed a significant increase in the titers and breadth of neutralizing antibodies in their serum,^44^ as well as a marked improvement in the proportion and duration of memory B cells. This indicates a stronger immune protective effect against emerging mutant strains.

This study underscores the importance of boosting our immunization against emerging VOCs such as Omicron, which has shown a strong ability to evade the immune system. Through the detection of WT/Omicron-specific MBC six months after initial immunization, it was demonstrated that Omicron poses a significant challenge to vaccine-induced immune memory. With a reduction in specific MBC, the immune system’s potential to fight against Omicron is diminished. However, the study also showed that a third booster dose significantly improves the levels of neutralizing antibodies and specific MBC, providing broad-spectrum and sustained protection against various variants. These findings support the need for booster immunization to enhance our immune response against emerging variants and to combat the ongoing COVID-19 pandemic.

There are still certain limitations worth noting in our research. Firstly, we recruited a relatively small number of volunteers, which may have an impact on the results, despite being statistically analyzed. In this case, the statistical analysis may not be accurate. Secondly, we did not conduct experimental verification of the deep-specific T-cell and B-cell immune response, so the actual clinical value of our results needs to be further explored. Thirdly, in the experiment analyzing the S protein-specific T-cell response, our results are relatively one-sided. The volunteers we recruited were infected with SARS-CoV-2 or vaccinated with inactivated vaccines, so the specific T-cell response is not limited to the S protein alone. We focused more on whether mutations can escape T-cell responses, so we selected the S protein with more mutation sites. Therefore, if a comprehensive evaluation of the T-cell induced by inactivated vaccines is required, other structural and non-structural proteins need to be included. Finally, inherent biases are unavoidable when conducting a retrospective study.

The aim of our study was to investigate the immune response of individuals with different immunological backgrounds after receiving inactivated vaccines. We conducted a thorough examination of the neutralizing antibodies, specific memory B and T-cell responses against various variants, with the intention of exploring the immune response induced by inactivated vaccines. Our research has also revealed that the variants have a weaker ability to escape T-cell response, providing a new avenue for vaccine development and targeted therapy in the future.

In conclusion, relying solely on a two-dose inactivated vaccine strategy for healthy individuals may not be sufficient to combat the emerging VOCs, particularly Omicron and its sub-lineages, which possess robust immune evasion capabilities. However, it is crucial to acknowledge that with the presence of strong immune memory, receiving a booster shot can significantly enhance the body’s ability to fend off each variant. This study highlights the importance of implementing a booster immunization approach, which can provide much-needed support to our immune systems as we continue to navigate the ever-evolving landscape of COVID-19. As such, it is imperative for healthcare providers and policymakers to prioritize and encourage booster shots as a critical component of our overall vaccination strategy.

## Materials and methods

### Cell lines

Vero cells (ATCC, CCL-81) and HEK293T/F cells (ATCC, CRL-3216) were cultured in Dulbecco’s Modified Eagle’s Medium (DMEM) supplemented with 10% fetal bovine serum (FBS). The cell line was cultured in 37 °C incubator with 5% CO_2_.

### Viral stocks

The SARS-CoV-2 wild-type strain CN01 was isolated from a patient in China during the early phase of the COVID-19 endemic. The SARS-CoV-2 variants of concern (VOC) beta (B.1.351 lineage), was isolated from a patient traveling back from South Africa; VOC gamma (P.1 lineage) was isolated from a person in Brazil; VOC delta (B.1.617.1 lineage) was isolated from a traveler with from India; and the newly emerged VOC Omicron (B.1.1.529 lineage), was isolated from a patient in Hong Kong and now preserved in SinoVac Biotech Ltd. All viruses were purified by standard plaque assay, and the clones of each passage were sequenced, and then inoculated into vero cells.

### Facility and ethics statements

All experiments associated with live SARS-CoV-2 viruses were performed in Biosafety Level 3 (BSL-3) laboratories in the Animal experiment Committee Laboratory Animal Center, Beijing Institute of Microbiology and Epidemiology.

### Human sera

The serum sample were taken from healthy individuals with no history of COVID-19 and recovered patients with previous infections of COVID-19. All volunteers received two doses or three doses CoronaVac (Sinovac) inactivated vaccine specific against SARS-COV-2 and signed the informed consent form.

### Study design and participants

We did a multi-center, open-label, non-randomized clinical trial to evaluate the safety, tolerability, and immunogenicity of inactivated SARS-CoV-2 vaccine (CoronaVac, Sinovac Life Sciences, Beijing, China) in adults with previously confirmed SARS-CoV-2 infection. Eligible participants were adults with previous SARS-CoV-2 infection history and healthy adults. Adults with SARS-CoV-2 infection were confirmed by positive nucleic acid for SARS-CoV-2 in pharyngeal swabs or sputum. Healthy adults who did not have SARS-CoV-2 infection were confirmed by negative results of serum specific IgM and IgG antibodies or negative nucleic acid for SARS-CoV-2 in pharyngeal swabs or sputum, and a clear chest CT image with no evidence of lesions in the lungs at the time of screening. The key exclusion criteria included s aged younger than 18 years or older than 60 years, axillary temperature of more than 37·0°, and history of allergy to any vaccine component. A complete list of exclusion criteria is listed in the supplementary data. All participates were recruited from the West China Hospital Sichuan University, Chengdu and Three Gorges Hospital Chongqing University, Chongqing. All participates received two doses of vaccine at 4-week intervals. The protocol and informed consent were approved by the Institutional Review Board of West China Hospital, Sichuan University (Chengdu, China; project identification code: 2021–495). All patients provided written informed consent. The study was done in accordance with the requirements of Good Clinical Practice of China and the International Conference on Harmonisation.

### Adverse reactions

Most adverse reactions were mild (grade 1) and moderate (grade 2) in severity; no participates had grade 3 adverse reactions. The most common injection site adverse reaction was pain, which was reported in 19 (32.2%) vaccine recipients. Pain was reported in 12 (38.7%) participants in the previous infection group and 7 (25.0%) participants in the high dose group. The most commonly reported systematic adverse reactions overall were fatigue (5 [8.5%]), which was reported in 2 (6.5%) participants in the previous infection group and 3 (10.7%) participants in the high dose group.

### Protein expression and purification

The plasmids encoding the full-length spike (S) protein (residues 1–1028) of wild-type SARS-COV-2 (GenBank: MN908947) were used as templates for the construction of S of the variants of concern by overlapping PCR in our lab. The variants of concern include B.1.351 (with mutations of 242–244 del, L18F, D80A, D215G, K417N, E484K, N501Y, D614G and A701V), P.1 (with mutations of L18F, T20N, P26S, D138Y, R190S, K417T, E484K, N501Y, D614G, H655Y and T1027I), B.1.617.2 (with mutations of T19R, G142D, 156del, 157del, R158G, L452R, T478K, D614G, P681R, D950N) and B.1.1.529 (with mutations of A67V, Δ69–70, T95I, G142D, Δ143–145, Δ211, L212I, ins214EPE, G339D, S371L, S373P, S375F, K417N, N440K, G446S, S477N, T478K, E484A, Q493K, G496S, Q498R, N501Y, Y505H, T547K, D614G, H655Y, N679K, P681H, N764K, D796Y, N856K, Q954H, N969K, L981F). Two proline substitutions at residues 986 and 987, ‘GSAS’ substitutions at the S1/S2 furin cleavage site (residues 682–685) and a C-terminal T4 fibritin foldon domain were remained in all the full-length S gene constructs to stabilize the trimeric conformation of S protein and facilitate the protein expression. A C-terminal twin-strep-tag II were attached to all the constructs to facilitate the protein purification. The plasmids described above were transiently transfected into HEK293 F cells grown in suspension in a 37 °C humidified incubator with 5% CO_2_, rotating at 130 rpm. After transfection for 72 h, the tangential flow filtration cassette is used to harvest and concentrate the supernatant and exchange the protein into the binding buffer. The protein of interest was purified by affinity chromatography using resin attached with streptavidin and further dialyzed into a buffer containing 20 mM Tris pH 8.0 and 200 mM NaCl.

### Collection of human peripheral blood mononuclear cells

Volunteer recruitment and blood draw were approved by West China School of Medicine. Study participants including 31 recovered patients with prior infections 12 month ago and 28 healthy individuals receiving two doses of CoronaVac inactivated vaccines donated blood at 0 wk, 1st wk, 2nd wk, 4th wk, 5th wk, 6th wk, 8th wk and 6th mth after first dose. The female:male ratio was 23:36 and the age range is 20–57. Human peripheral blood mononuclear cells (PBMCs) were isolated, aliquoted, and stored in liquid nitrogen after collection of peripheral blood for subsequent experiment.

### Authentic virus neutralization assay

The serum samples were first inactivated by incubation at 56 °C for 30 min. Heat-treated serum samples were diluted by serial dilution from 1:4 with DMEM in two-fold steps and mixed with a virus suspension containing 100 TCID50 and incubated at 36.5 °C for 2 h. The mixture was added to a 96-well plate seeded with confluent Vero cells in advance and incubated in incubator with humidified 5% CO_2_ at 36.5 °C for another 5 days. The Cytopathic effect (CPE) of each well was observed and recorded by three different individuals under microscopes, and the related dilutions were calculated using the Reed–Muench method to obtain the neutralization titer of samples.

### Pseudovirus neutralization assay

In order to obtain the pseudotyped viruses, 293T cells were first transfected with the constructed plasmids encoding the full-length spike (S) protein of wild-type and VOC (B.1.1.7, B.1.351, P.1, B.1.617.2 and B.1.1.529) SARS-CoV-2. The transfected 293T cells were incubated with VSV G pseudotyped virus (G*ΔG-VSV) at a multiplicity of infection (MOI) of 4 for 5 ho. The infected cells were then added with complete culture medium after washing with PBS. After incubation for another 24 h, the SARS-CoV-2 wild-type and VOC pseudoviruses were produced, aliquoted, and stored for the In vitro pseudotyped virus neutralization assay. The plasma samples were diluted with DMEM from 1:10 with six additional threefold serial dilutions and mixed with the SARS-CoV-2 pseudoviruses at 37 °C for 1 h. The mixture was added to a Huh-7 cells and incubated in incubator with humidified 5% CO_2_ at 36.5 °C for another 24 h. After that, the luciferase luminescence (RLU) of each well was measured with a luminescence microplate reader. The neutralization percentage was calculated as following: Inhibition (%) = [1- (sample RLU- Blank RLU) / (Positive Control RLU-Blank RLU)] (%).

### S/RBD-specific B cell

The stored Human peripheral blood mononuclear cells (PBMCs) were first treated with following steps. The PBMCs were quickly thawed in a 37 °C water bath. The suspensions were centrifuged at 500 × *g* for 5 min to remove the supernatant. The PBMCs were resuspended with 2% FACS buffer (phosphate buffered saline containing 2% fetal bovine serum). The PBMCs washed with 2% FACS buffer were filtered by Corning (Falcon) 100 μm cell strainers. After the pretreatment, the PBMCs were incubated with human Fc block (BioLegend) in the dark at 4 °C for 15 min. The PBMCs were then incubated with APC-cy7 anti-human CD3 (BioLegend), APC anti-human CD20 (BioLegend), FITC anti-human CD19 (BioLegend) and spike (S) protein-biotin in the dark at 4 °C for 30 min. The cells were then incubated with PerCP/Cyanine5.5 Streptavidin (BioLegend) and PE Streptavidin (BioLegend) in the dark at 4 °C for 30 min.

### Enzyme-linked immunosorbent assay (ELISA)

The SARS-CoV-2 ELISA assay was performed. Briefly, we used an indirect ELISA with different antigens. 96-well plates (Corn)were first coated with antigen (10 μg/ml) in phosphate buffered saline (PBS) overnight at 4 °C, and then blocked with 2% bovine serum albumin (BSA) in PBS at room temperature for 2 h. After that, the serum samples were serially diluted 1:3 in PBS for eight dilutions in total, with maximum concentration 1:10, and then incubated with antigen for 1 h at room temperature. After incubation, HRP-conjugated second antibody (Thermo Fisher Scientific) was added for visualization. The area under the curve (AUC) was calculated by analysis using PRISM software to evaluate the antigen-binding capacity.

### ELISPOT assay

The antigen-specific immune response for T cells in PBMCs was detected by ELISPOT using Human IFN-γ ELISpotPLUS kit (MABTECH, Product Code: 3410-4APW-10). The pre-coated plates were washed with sterile PBS (200 μl/well) for 4 times and added with medium (200 μl/well) containing 10% of the same serum as used for the cell suspensions (5 × 10^5^ cells/well) and incubated at room temperature for 30 min. After removing the medium, the spike (S) protein was added as a stimulate followed by the treated cell suspension. The mAb CD3-2 at a dilution of 1:1000 was used as a positive control for cytokine production. The plates were incubated in a 37 °C humidified incubator with 5% CO_2_ for 48 h. After that, the cells were removed and the plates were washed with PBS (200 μl/well) for 5 times. The detection antibody IFN-γ-II-biotin (diluted to 1 μg/ml in 0.5% Facs buffer) was added in plates (100 μl/well) and incubated at room temperature for 2 h. The Streptavidin-ALP (1:1000) was diluted in 0.5% FACS buffer and added in plates (100 μl/well). After incubation, the spots were visualized with the substrate solution (BCIP/NBT-plus). The IFN-γ secretion was quantified using an ELISpot reader.

## Supplementary information

supplemental material

## Data Availability

All resources used in this study are availability from corresponding authors upon reasonable request.
