# Supplementary material for: Comparing T- and B-cell responses to COVID-19 vaccines across varied immune backgrounds
Source: Signal Transduct Target Ther. 2023 May 4;8:179. doi: 10.1038/s41392-023-01422-7 (PMC10157553; doi:10.1038/s41392-023-01422-7)
Supplement: Supplementary file 1 — supplemental material [file 41392_2023_1422_MOESM1_ESM.docx]

Supplementary Materials for

**Immunological profile of previously infected individuals after vaccination**

**Author:** Zhen Cui, Wenxin Luo, Ruihong Chen, Yalun Li, Shuo Liu, Zhoufeng Wang, Yong Liu, LeiLei Feng, Zijing Jia, Ruixin Cheng, Jun Tang, Huawen Liu, Weijin Huang, Yanjun Zhang, Xiangxi Wang and Weimin Li

Correspondence: W.M.L. (Email: [weimi003@scu.edu.cn](mailto:weimi003@scu.edu.cn)) or X.W. (Email: [xiangxi@ibp.ac.cn](mailto:xiangxi@ibp.ac.cn) ) or H.W.L (Email: [liuhw008@163.com](mailto:%20liuhw008@163.com) )

**This PDF file includes:**

Figure. S1 to S5

**Fig. S1.**

**
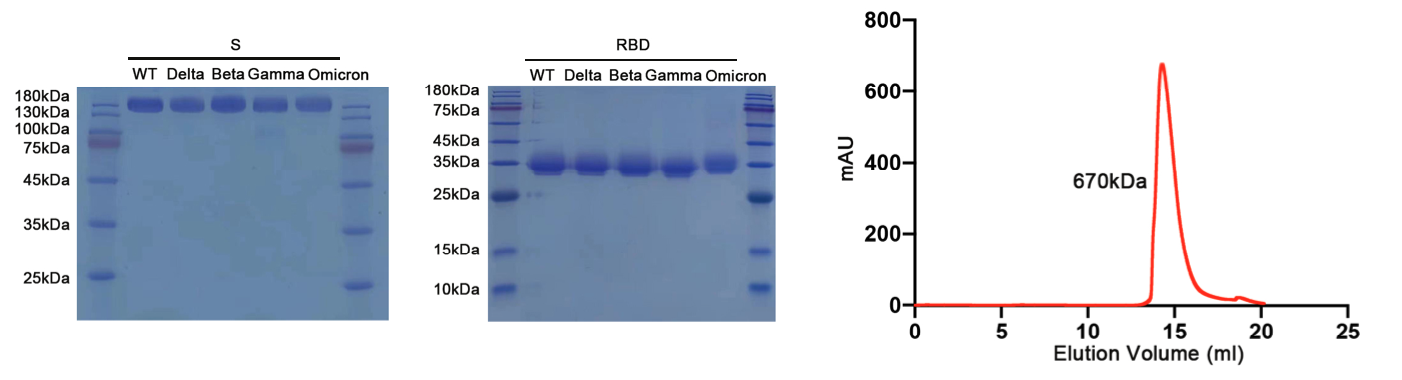
**

**Fig. S1. Purification and characterization of S trimer and RBD.**

SDS-PAGE analysis of the Wild-type, Delta, Beta, Gamma and Omicron S trimer and RBD. Gel filtration profile of the affinity-purified Omicron S trimer.

**Fig. S2.**


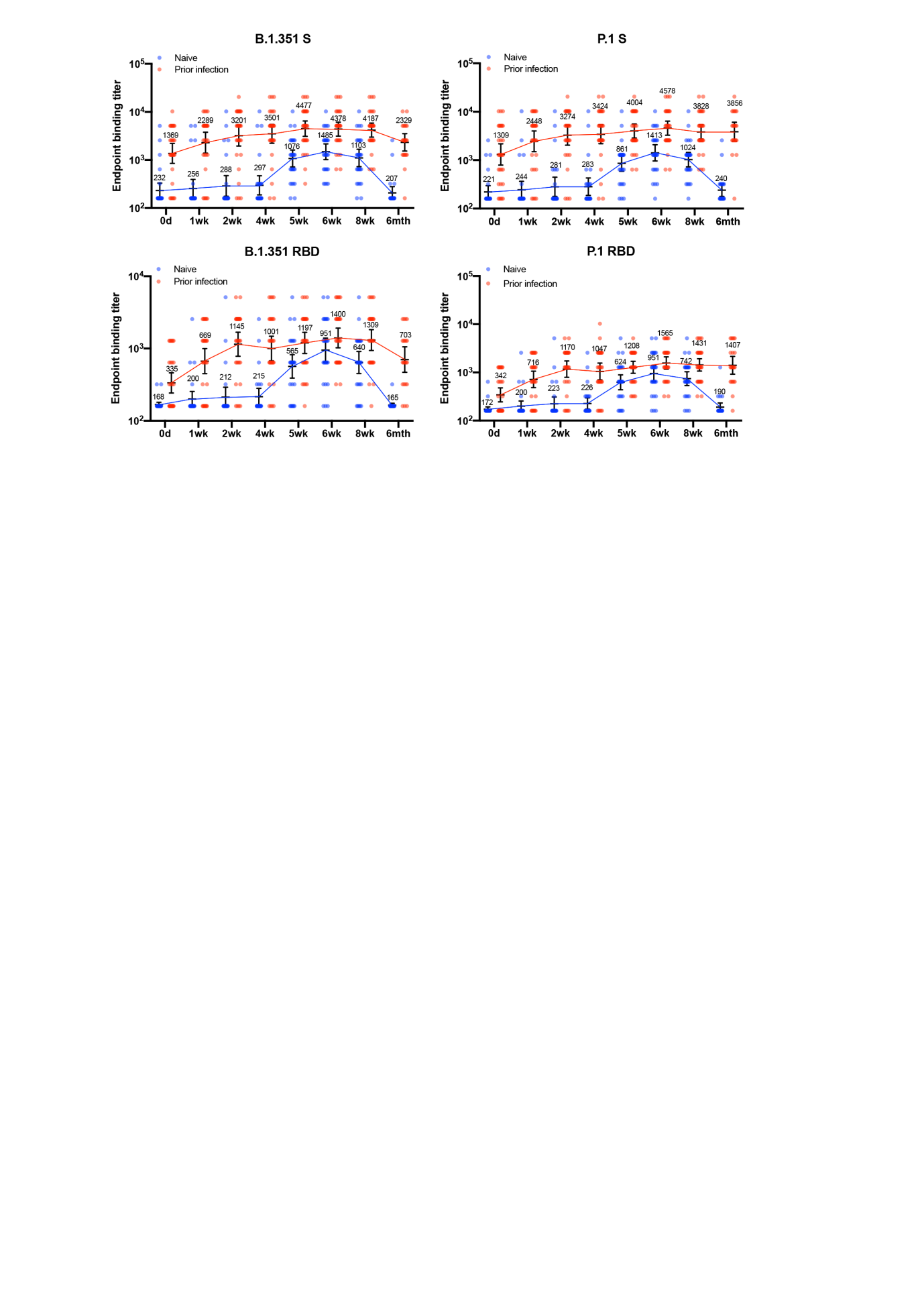


**Fig. S2. S protein-specific antibody responses**

Endpoint antibody responses specific to the spike protein (S) of B.1.351(left), and P.1(right) VOCs from recovered and naïve vaccinees at eight timepoints. Endpoint antibody responses specific to the receptor binding domain (RBD) of B.1.351(left), and P.1(right) VOCs from recovered and naïve vaccinees at eight timepoints

**Fig. S3.**


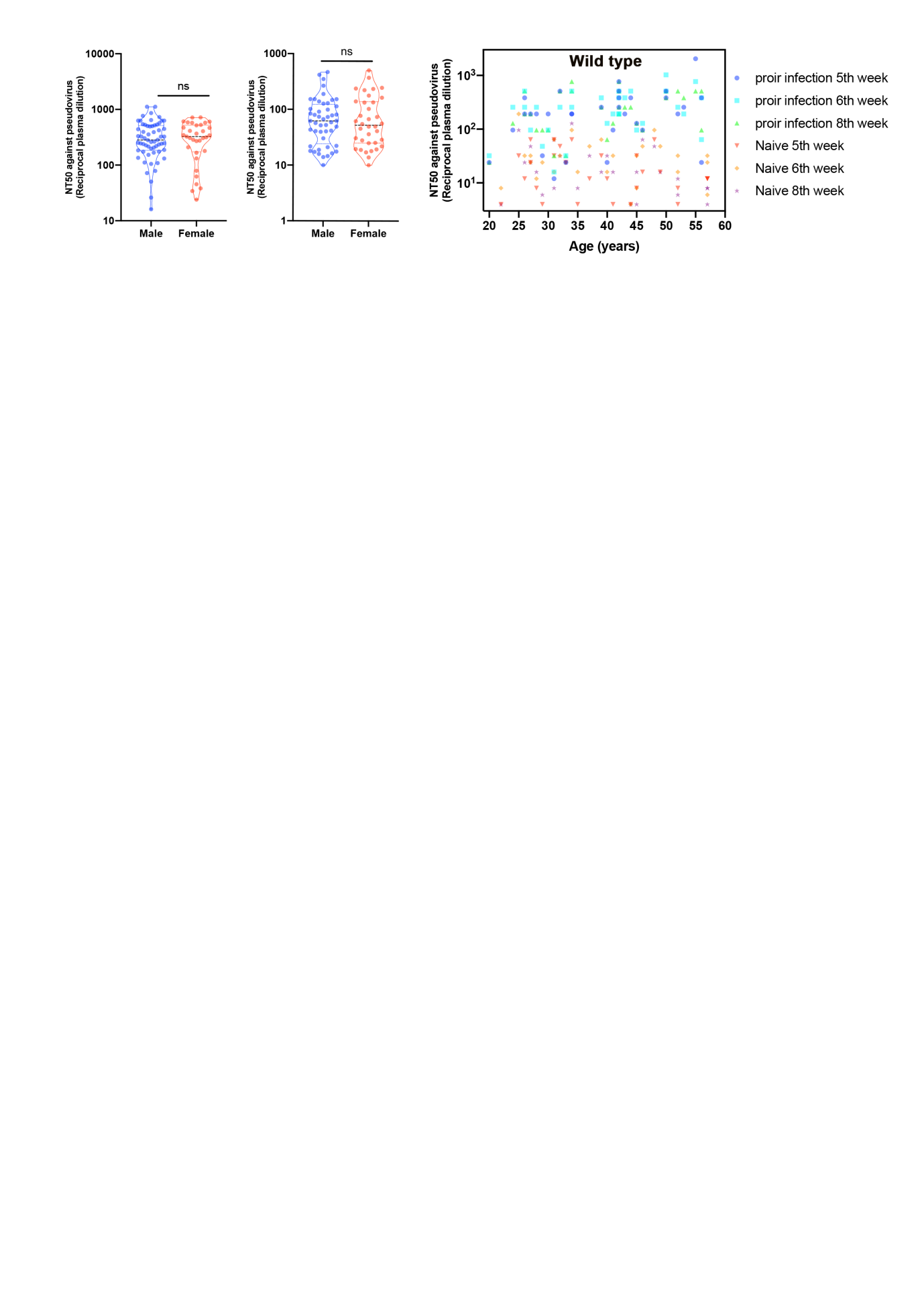


**Fig. S3. Correlation of neutralizing antibodies with age and sex.**

Neutralization of wild-type SARS-CoV-2 pseudovirus is compared female with male. Bar and error bars indicate mean and standard deviation. Neutralization of wild-type SARS-CoV-2 pseudovirus is plotted against age.

**Fig. S4.**


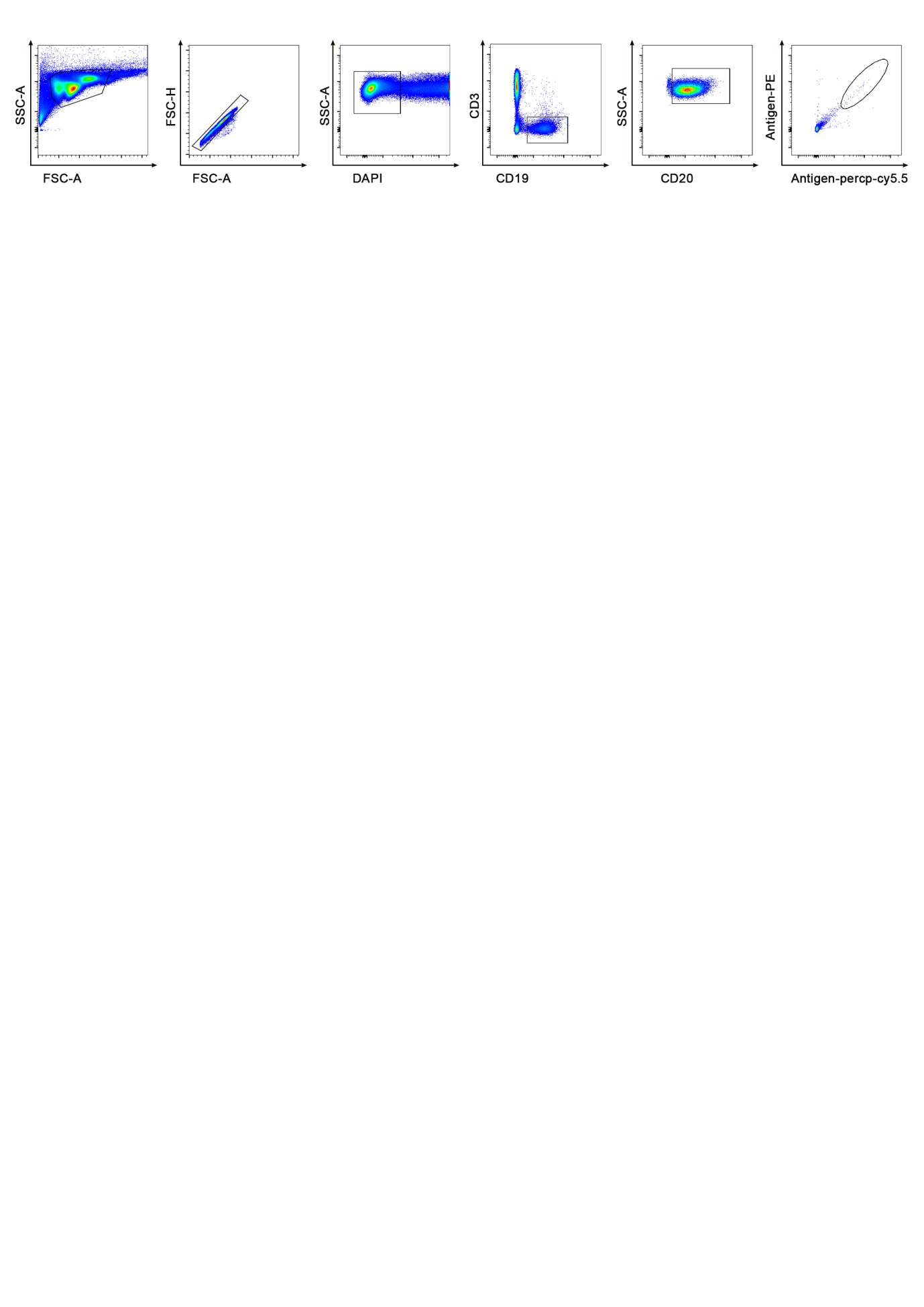


**Fig. S4. Flow Cytometry Analysis Strategy**

Representative gating strategy for identification of proliferating dual S-percp-cy 5.5 and phycoerythrin (PE)-S-binding B_mem_ cells

**Fig. S5.**


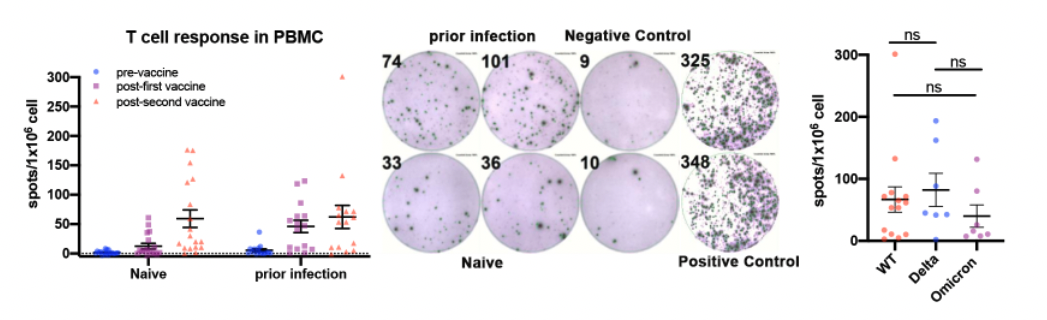


**Fig. S5. The antigen-specific immune response of T cell**

The antigen-specific immune response for T cells in PBMCs from study participants detected by ELISPOT. Mid: Representative IFN-γ ELISpot responses for study participants, negative control; positive control. Left: Comparative IFN-γ ELISpot spot forming units (SFUs) per 10^6^ peripheral blood mononuclear cells (PBMCs) in individuals with prior infection and naïve.
